# Planning effective mental healthcare in prisons: findings from a national consultation on the care programme approach in prisons

**DOI:** 10.1192/bjo.2021.533

**Published:** 2021-06-18

**Authors:** Jemini Jethwa, Kate Townsend

**Affiliations:** The Royal College of Psychiatrists

## Abstract

**Aims:**

The Care Programme Approach (CPA) can be an effective tool in coordinating the care and treatment needs of people with mental illness and learning disabilities. Within prisons settings, the CPA has been poorly implemented and the principles underpinning this approach have been lost. The aim of this research was to look at the key themes identified as part of a consultation process to develop quality guidance on planning effective mental healthcare in prisons in relation to the CPA.

**Method:**

The consultation exercises included telephone interviews and hosting a national consultation event to represent the views of prisons nationally. It was conducted by the Quality Network for Prison Mental Health Services, a quality improvement initiative organised by the Royal College of Psychiatrists’ Centre for Quality Improvement.

**Result:**

The results derived from the consultation process indicates that CPA in prisons is inconsistently adopted and that there is lack of confidence in the process from prison mental health teams, particularly with how to engage community mental health teams.

**Conclusion:**

This concludes that there is a substantial need for standardisation and consistency in the application of the CPA process within prisons, for the purposes of enhanced care delivery, greater continuity of care, and improved patient outcomes. The Quality Network for Prison Mental Health Services used the findings from this consultation to produce a national guidance document on planning effective mental healthcare in prisons, which can be accessed for free by all prison mental health teams.

